# Somatosensory Evoked Potentials in Spinocerebellar Ataxia Type 3 and Type 10

**DOI:** 10.1007/s12311-026-01998-0

**Published:** 2026-04-17

**Authors:** Léo Coutinho, Otto Jesus Hernandez Fustes, Carlos Henrique Ferreira Camargo, Gabriel Abrahão Stoliar, Francisco Manoel Branco Germiniani, Salmo Raskin, Tetsuo Ashizawa, Cláudia Suemi Kamoi Kay, Paulo José Lorenzoni, Renata Dal-Prá Ducci, Rosana Herminia Scola, Hélio A. Ghizoni Teive

**Affiliations:** 1 Department of Internal Medicine, Neurological Diseases Group, Postgraduate Program in Internal Medicine, Federal University of Paraná, Rua General Carneiro, 181. Alto da Glória, Curitiba, PR 80060-900 Brazil; 2 Department of Internal Medicine, Hospital de Clínicas, Service of Neuromuscular Disorders, Division of Neurology, Federal University of Paraná, Curitiba, PR Brazil; 3 Department of Internal Medicine, Hospital de Clínicas, Movement Disorders Unit, Division of Neurology, Federal University of Paraná, Curitiba, PR Brazil; 4 Genetika - Genetic Counseling Center and Laboratory, Curitiba, PR Brazil; 5 Weill Cornell Medicine at Houston Methodist Hospital, Houston, TX USA

**Keywords:** Movement disorders, Ataxia, Spinocerebellar ataxias, Neurophysiology, Somatosensory evoked potentials

## Abstract

Spinocerebellar ataxias (SCAs) are heterogeneous neurodegenerative disorders with variable phenotypes. Somatosensory evoked potentials (SSEPs) provide insights into the integrity of the dorsal-lemniscal pathway. This study aimed to evaluate SSEP findings in patients with SCA3 and SCA10 and compare their neurophysiological profiles. We conducted a cross-sectional study including 20 SCA3 patients and 20 SCA10 patients under follow-up at a tertiary ataxia clinic in Southern Brazil. Clinical assessment comprised demographic, genetic, and phenotypic characterization, including the Scale for the Assessment and Rating of Ataxia (SARA). SSEPs were recorded bilaterally after median and tibial nerve stimulation, assessing latencies and amplitudes of N9, N20, N21, and P40 potentials, as well as interpotential intervals. Examinations were classified as normal or abnormal, and abnormalities were categorized. Abnormal SSEPs were detected in 18 SCA3 patients (90%) and in 3 SCA10 patients (15%) (*p* < 0.001). In SCA3, abnormalities predominantly involved central generators. In contrast, most SCA10 patients exhibited normal SSEPs, with only a few isolated cortical responses and interval abnormalities. No significant associations were found between SSEP abnormalities and demographic, clinical, imaging, or genetic variables in either group. SCA3 shows widespread somatosensory dysfunction, whereas SCA10 patients, particularly in Southern Brazil, show preserved SSEPs consistent with pure cerebellar ataxia. These findings highlight distinct neurophysiological signatures in SCAs, reinforcing their heterogeneity and supporting SSEPs as candidate markers for differential characterization, requiring validation.

**Supplementary Information:**

The online version contains supplementary material available at 10.1007/s12311-026-01998-0.

## Introduction

Spinocerebellar ataxias (SCAs) are a heterogeneous group of autosomal dominant neurodegenerative disorders characterized by progressive cerebellar ataxia accompanied by variable involvement of extracerebellar structures, including the brainstem, spinal cord, basal ganglia, and somatosensory pathways. Although cerebellar dysfunction is the clinical hallmark of these conditions, the phenotypic diversity observed across SCAs reflects the extent of multisystem neurodegeneration rather than cerebellar impairment alone [1–3].

More than 50 genetically distinct SCAs have been identified, each associated with specific patterns of neurodegeneration that translate into clinical phenotypes. As a result, patients with different SCA subtypes may present distinct clinical, imaging, and neurophysiological profiles, reflecting the underlying distribution of neurodegeneration, and different phenotypes exist among patients that share the same genotype [2, 3].

Spinocerebellar Ataxia type 3 (SCA3), the most common form of SCA worldwide, exemplifies this pattern of neurodegeneration. Caused by a CAG repeat expansion in the *ATXN3* gene, SCA3 is characterized not only by cerebellar ataxia but also by widespread neurodegeneration. Neuropathological and neuroimaging studies have consistently demonstrated degeneration affecting the dorsal-lemniscal pathway and somatosensory cortical regions, providing a biological substrate for potential impairment of somatosensory conduction in these patients [4–10].

Spinocerebellar ataxia type 10 (SCA10), caused by an intronic ATTCT repeat expansion in the *ATXN10* gene, represents a distinct model of phenotypic variability across populations. While patients from Hispanic-Amerindian cohorts frequently present with epilepsy and additional extracerebellar features, studies conducted in Southern Brazil have consistently demonstrated a predominance of pure cerebellar ataxia with relatively limited extracerebellar involvement [11–13]. Recent multimodal neuroimaging studies carried out among Hispanic patients have demonstrated that SCA10 is associated not only with cerebellar atrophy but also with increased functional connectivity within cerebellar and sensorimotor networks, suggesting the presence of network-level dysfunction and possible maladaptive compensatory mechanisms beyond structural degeneration [14]. In contrast, neuroimaging studies conducted in the Southern Brazilian population demonstrated relative preservation of extracerebellar structures [7, 8]. This population-specific phenotype suggests a more restricted pattern of neurodegeneration, potentially sparing key components of the somatosensory pathways, and raises important questions regarding the extent to which central sensory pathways are functionally preserved in SCA10 across different populations.

Somatosensory evoked potentials (SSEPs) provide a non-invasive and objective assessment of the functional integrity of the dorsal-lemniscal pathway, evaluating both peripheral and central sensory conduction [15–17]. Previous studies exploring SSEP abnormalities in SCA3 are limited and have reported heterogeneous findings, generally indicating predominant involvement of central conduction pathways, particularly affecting cortical responses such as the P40 potential [18–21]. Given the contrasting patterns of extracerebellar neurodegeneration between SCA3 and SCA10, we hypothesized that these disorders would exhibit distinct SSEP profiles, with more frequent and severe abnormalities in SCA3 and relative preservation in SCA10.

In this context, the present study aimed to investigate somatosensory pathway involvement in SCA3 and SCA10 using SSEPs. By directly comparing these two genetically and clinically distinct ataxias, we sought to determine whether SCA10 exhibits relative preservation of central sensory conduction when contrasted with the more multisystemic involvement observed in SCA3.

## Methods

This observational study was conducted and reported in accordance with the *Strengthening the Reporting of Observational Studies in Epidemiology* (STROBE) *Statement*, ensuring transparency and methodological rigor in the design and reporting of results [22].

We conducted a cross-sectional study of SCA3 and SCA10 patients under follow-up at the Ataxia Outpatient Clinic in Hospital de Clínicas (Curitiba, Paraná, Brazil) from 2021 to 2025. Patients aged ≥ 18 years with genetically confirmed SCA3 or SCA10 attending their outpatient appointments at the Ataxia Clinic were invited to participate in this research.

We excluded patients based on the following criteria: (1) Peripheral neuropathy that has been proven to be unrelated to the ataxia condition; (2) Cognitive impairment that could render the SSEP exam impossible; (3) Refusal or withdrawal from participating in the research; (4) Patients with an electrical device that could present a malfunction during electrical stimulation or that could interfere with the neurophysiological data (e.g., pacemaker); and (5) Patients who did not tolerate the electrical stimuli.

This study was reviewed and approved by our institutional ethics review board (Number 74783421.5.0000.0096). All patients provided written informed consent to take part in this study.

### Clinical Assessment

The patients underwent a clinical assessment and comprehensive neurological examination to gather information regarding the following epidemiological, clinical, and genetic variables: sex, age, age of onset, disease duration, clinical phenotype, and expansion (CAG or ATTCT) length.

The neurological examination included assessment of muscle strength as measured by the Medical Research Council (MRC) scale [23], evaluations of deep tendon reflexes and pathological reflexes, evaluation of Parkinsonian features, assessment of muscle tone, assessment of all modalities of sensation (touch, temperature, vibration, and proprioception), and Romberg’s test. Ataxia severity was measured by the *Scale for the Assessment and Rating of Ataxia* (SARA) [24].

To support the phenotyping process and evaluate conditions that could lead to patient exclusion, we reviewed medical records for information on magnetic resonance imaging (MRI) and laboratory tests. Brain MRI scans were retrospectively reviewed using axial and sagittal T1-weighted sequences. MRI findings were qualitatively assessed (normal vs abnormal), and global cortical atrophy was further graded using the Pasquier scale (Global Cortical Atrophy, GCA), an ordinal visual rating scale ranging from 0 (no atrophy) to 3 (severe atrophy), based on sulcal widening and ventricular enlargement [25]. All evaluations were performed by the same examiner, who was blinded to the genetic diagnoses.

Peripheral neuropathy classification was supported by retrospective review of available nerve conduction studies (NCS), which were not systematically performed in all patients, and by clinical evaluation. This information was incorporated into the phenotyping process.

The phenotypic classes included in our study were determined based on prior studies conducted in our population [4, 12]. All clinical assessments were performed by the same examiner.

### SSEPs

Following the clinical assessment, patients had their SSEP scheduled at their convenience. Patients were accommodated in a silent room with minimal luminosity to ensure maximum relaxation and minimal external interference. The room temperature was kept between 23 and 25ºC, and limb temperature was maintained > 32ºC, with heating if necessary [16, 26]. Due to the need for real-time access to the patients’ files, necessary to register the SSEP results, as well as clinical data and patient identification during acquisition and reporting, blinding of the neurophysiological examiner to the genetic diagnosis was not feasible. To mitigate selection bias, a random subset of exams was separated for independent review (5 random patients from each SCA group).

The SSEPs were performed using a 4-channel device with a *Nihon-Kohden Neuropack*® software, using the standard test configurations and reference values established by the *American Clinical Neurophysiology Society* (ACNS) and *International Federation of Clinical Neurophysiology* (IFCN) [16, 26].

Electrode placement was performed in accordance with the ACNS guidelines, including the EP, C5s, CP, Fz, AC, PF1-PF2, T12s, and IC electrodes. Electrode impedance was kept below 5kΩ [17, 26]. Recording bandpass was set between 0.1 and 200 Hz. Selected montages were EP-Fz and CP-Fz for the median nerve and T12s-IC and CP-Fz for the tibial nerve. For upper-limb SSEP, we performed transcutaneous median nerve stimulation at the wrist, using surface electrodes placed over the nerve between the palmaris longus and flexor carpi radialis tendons. We delivered short-lasting square-wave pulses of 0.2 ms duration with a repetition rate of 2-5 Hz and analysis time of 100 ms. Stimulus intensity was sufficient to produce a visible thumb twitch, avoiding additional discomfort. At least 1000 stimuli were obtained for averaging and reliable responses [16, 26]. For lower-limb SSEP, we performed transcutaneous tibial nerve stimulation at the ankle, with electrodes positioned posterior to the medial malleolus. We delivered short-lasting square-wave pulses of 0.2 ms duration with a repetition rate of 1-2 Hz. Stimulus intensity was sufficient to elicit a visible plantar flexion of the toes or foot. At least 1000 stimuli were obtained for averaging and reliable responses [16, 26].

For this study, we assessed the N9 potential, N20 potential, and N9-N20 intervals for the median nerve stimulation bilaterally, and the N21 potential, P40 potential, and N21-P40 intervals for the tibial nerve stimulation. We selected these potentials for our study protocol because they can capture both peripheral and central structures of the dorsal-lemniscal pathway, are easily obtainable, and are less prone to interference from electrical noise. We measured the potentials' latencies and amplitudes, as well as the latencies of the interpotential intervals. These numerical variables were compared to the established IFCN normal ranges to determine exam normality (Suppl. Table 1). Latencies for the N21 and P40 potentials were interpreted considering patient height. We characterized exam normality dichotomously (normal vs abnormal), and all neurophysiological findings were computed at the patient level, with each variable considered abnormal if at least one side showed alteration. In accordance with IFCN guidelines, side-to-side differences were accounted for in determining exam abnormalities [16].

After this primary analysis, for further quantitative analyses of SSEP, latencies and amplitudes values were averaged between sides when both responses were present. In cases where a response was absent on one side, the value from the preserved side was used. Patients with bilateral absence were excluded from parameter-specific analyses.

### Statistical Analysis

All numerical variables were tabulated in Microsoft Excel® and expressed in terms of means ± standard deviations for normally distributed variables and median [interquartile intervals] for non-normally distributed variables. We performed the statistical analysis using the open-source software R (version 4.5.1) [27]. The *p*-values indicating a statistically significant difference were fixed at 0.05 (5%).

We assessed the distribution of numerical variables using the Shapiro–Wilk test and compared group differences using a Student’s t-test for normally distributed variables and a Mann–Whitney U test for non-normally distributed variables. For the non-parametric tests, we also calculated effect sizes with Cliff’s delta (δ). Values for Cliff delta were fixed in < 0.33 for a small effect size, 0.33–0.47 for medium effect size, and > 0.47 for a large effect size. Post-hoc correlation analyses were performed to assess the relationship between neuroimaging findings and neurophysiological abnormalities. Spearman’s rank correlation coefficient (ρ) was used to evaluate the association between Global Cortical Atrophy (GCA) scores and the presence of SSEP abnormalities. These analyses were conducted separately for the SCA3 and SCA10 groups.

Outliers were identified using the interquartile range (IQR) method (Tukey rule), defined as values below Q1 − 1.5 × IQR or above Q3 + 1.5 × IQR. Additionally, sensitivity analyses were performed when applicable to assess the robustness of the findings. These included analyses after exclusion of outliers identified by the IQR method, as well as analyses restricted to patients with available and complete data for specific variables. The results of these analyses were compared to the primary findings to evaluate the stability and consistency of the observed effects. Categorical variables were compared among groups using a Chi-squared test with Yates' correction and a Fisher’s exact test. Bonferroni correction was used for multiple comparisons.

SSEP findings were classified as normal or abnormal, and diagnostic performance metrics (sensitivity, specificity, and odds ratios with 95% confidence intervals) were calculated using standard 2 × 2 contingency tables, considering SCA3 as the target condition.

In order to assess correlations between the epidemiological, clinical, and genetic data and the presence of abnormalities in the SSEPs we performed a stepwise approach: 1) We performed nonparametric tests to compare the distribution of the variables between the groups with normal SSEPs and abnormal SSEPs, first for the SCA3 patients, then for the SCA10 patients; 2) As an exploratory secondary analysis, we performed a penalized logistic regression model (LASSO) with crossed validation and Bootstrapping resampling (B = 1000), generating penalized coefficients and their 95% confidence intervals (95%CI), selection frequencies, and empirical *p* values. Missing data in the predictive variables were handled through multiple imputations, generating m = 10 imputed bases using the chained equations (MICE) method, thereby maintaining statistical power and reducing the exclusion of cases.

## Results

During this research project, 26 patients with SCA3 and 30 with SCA10 were invited to participate. In the SCA3 sample, six patients were excluded under the following circumstances: 4 patients declined to take part in the research as they were living in different states, with logistical difficulties to follow the research protocol, one patient was excluded as a result of a peripheral neuropathy presumably unrelated to her genetic diagnosis (presumed HIV-related), and one patient was excluded as a result of severe dementia. This resulted in a final sample of 20 SCA3 patients from 15 unrelated families.

As for the SCA10 group, 10 patients were excluded as follows: 5 patients declined to take part in the research as they were living in different states, three patients had their SSEP exams scheduled but missed their appointments and failed to respond further contacts to reschedule, one patient was excluded as a result of severe dementia, one patient did not tolerate the electrical stimuli with minimal intensity. The final SCA10 sample comprised 20 patients from 9 unrelated families.

MRI was available for the entire SCA3 and SCA10 sample. In the SCA3 group, 18 patients had mild cerebellar atrophy, 1 had a normal exam, and 1 had pontine, cortical, and cerebellar atrophy. In the SCA10 group, 16 patients presented with mild cerebellar atrophy; 3 patients had normal exams, and one patient had a small focus of gliosis in the left cerebellar hemisphere, which was considered incidental. In the SCA3 group, GCA scores were 0 in 9 patients (45%), 1 in 9 (45%), and 2 in 2 (10%) (median = 1). In the SCA10 group, GCA scores were 0 in 10 patients (50%), 1 in 6 (30%), and 2 in 4 (20%) (median = 0).

In the SCA3 group, 3 patients had NCS, with evidence of peripheral neuropathy, while only one patient of the SCA10 group had NCS, with a normal exam.

Data regarding the distribution of demographic and clinical variables is summarized in Table 1. Comparisons of the distributions of sex, age, age of onset, disease duration, and SARA scores showed no statistically significant differences between SCA3 and SCA10 patients.

**Table 1:** Demographic and clinical distribution of the sample

Variable	SCA3 (*n* = 20)	SCA10 (*n* = 20)	*P* Value
Sex (Female)	10 (50%)	9 (45%)	1^*^
Age (Years)	44.5 [35.75 – 49.5]	44.5 [40.75 – 58.0]	0.297^**^
Age of onset (Years)	33.3 ± 10.0	32.4 ± 8.5	0.838^***^
Duration of disease (Years)	11 [6.0 – 17.5]	16.5 [11.5 – 23.75]	0.109^**^
SARA	13.0 [8.0 – 21.75]	10.0 [7.75 – 15.25]	0.400^**^
Expansion length (CAG/ATTCT)	71.1 ± 3.8 (*n* = 11)	1688.25 ± 601.24 (*n* = 20)	—
Phenotypic distribution	Pure cerebellar ataxia (*n* = 8) Ataxia + Pyramidal signs (*n* = 6) Ataxia + Peripheral neuropathy (*n* = 4) Mixed phenotype (*n* = 2)	Pure cerebellar ataxia (*n* = 15) Ataxia + epilepsy (*n* = 4) Ataxia with parkinsonism (*n* = 1)	0.054^#^ —

^*^Chi-Squared test **Mann–Whitney test ***Student’s t test

^#^ Fisher’s exact test for Pure cerebellar ataxia vs ataxia + other symptoms

*SARA* Scale for the Assessment and Rating of Ataxia

Data on repeat lengths were available for 11 patients in the SCA3 group and 20 in the SCA10 group. The SCA3 group presented a mean CAG repeat length of 71.1 (± 3.8) repetitions, while the SCA10 group presented a mean ATTCT repeat length of 1688.25 (± 601.24). In the SCA3 group, CAG repeat length was missing for 9 patients (45%). Sensitivity analysis revealed no statistically significant differences between patients with and without available CAG data in terms of age (*p* = 0.71), disease duration (*p* = 0.21), SARA scores (*p* = 0.62), or sex distribution (*p* = 0.37).

The phenotypic distribution of the SCA3 group showed a slight predominance of pure cerebellar ataxia (*n* = 8), followed by ataxia with pyramidal signs (*n* = 6), ataxia with signs of peripheral neuropathy (*n* = 4), and ataxia with mixed phenotype (*n* = 2), with variable combinations of pyramidal, peripheral, and extrapyramidal findings. In the SCA3 group, three out of four patients classified as having peripheral neuropathy had electrodiagnostic evidence of axonal polyneuropathy through NCS. One additional SCA3 patient, who did not have available electrodiagnostic data, was classified as having peripheral neuropathy based on clinical findings, including absent deep tendon reflexes and length-dependent sensory loss.

The SCA10 group presented a marked predominance of patients with pure cerebellar ataxia (*n* = 15), followed by ataxia with epilepsy (*n* = 4), and ataxia with Parkinsonism (*n* = 1).

Regarding the frequency of abnormal exams across groups, 18 patients in the SCA3 group (90%) had abnormal SSEP, whereas only three patients in the SCA10 group (15%) did. By comparing the frequencies of abnormalities between the groups, we detected a statistically significant difference (*p* < 0.001). A post-hoc power analysis demonstrated that the difference in SSEP abnormalities between SCA3 and SCA10 was associated with a very large effect size and high statistical power (power = 0.9997). Apart from unilateral absence of potential, no clinically relevant asymmetries beyond predefined abnormality thresholds were identified and incorporated into the classification of abnormal SSEP findings.

Overall SSEP abnormalities showed a sensitivity of 90% and specificity of 85% for identifying SCA3 over SCA10. The corresponding odds ratio was 51.0 (95% CI: 8.0–325), indicating a strong association between abnormal SSEP findings and SCA3.

The specific neurophysiologic abnormalities detected in both groups are summarized in Table 2. In the SCA3 group, we observed a predominance of the absence of the P40 potential, prolonged N9-N20 interval, and prolonged N21-P40 interval. In the SCA10 group, the few patients with abnormal exams exhibited absence of the P40 potential, reduced P40, delayed P40, prolonged N9-N20 interval, and delayed N20.

**Table 2:** Neurophysiological abnormalities per type of SCA

Potential	Type of abnormality	SCA3	SCA10	*P* Value	*P* – Adjusted*
N9	Absent	4	0	0.106	1.000
	Reduced	1	0	1.000	1.000
	Delayed	1	0	1.000	1.000
N9–N20	Prolonged	10	1	0.003	**0.047**
N20	Absent	5	0	0.047	0.660
	Reduced	2	0	0.487	1.000
	Delayed	4	1	0.342	1.000
N21	Absent	3	0	0.231	1.000
	Reduced	0	0	1.000	1.000
	Delayed	0	0	1.000	1.000
N21–P40	Prolonged	7	1	0.044	0.610
P40	Absent	16	2	< 0.001	**0.00023**
	Reduced	3	1	0.605	1.000
	Delayed	8	1	0.020	0.275

^*^Fisher’s exact test with Bonferroni correction

By comparing the frequencies of abnormalities between the groups, we detected statistically significant differences for prolonged N9-N20 interval (10 occurrences Vs 1, *p-*Adjusted = 0.047) and absent P40 potential (16 occurrences Vs 2, *p*-Adjusted = 0.00023). All other abnormalities did not reach statistical significance after Bonferroni correction.

In both groups, the comparison of the frequency of abnormalities per clinical phenotype showed no statistically significant difference. Subgroup analyses based on clinical phenotype were performed to assess the potential influence of phenotypic complexity on SSEP findings using a dichotomous classification (pure vs. complex phenotype). Within the SCA3 group, SSEP abnormalities were present in 11 of 12 patients with complex phenotypes and in 7 of 8 patients with pure phenotypes, with no significant difference between subgroups (*p* = 1.00). Similarly, within the SCA10 group, SSEP abnormalities were observed in 1 of 5 patients with complex phenotypes and in 2 of 15 patients with pure phenotypes, also without a significant difference (*p* = 1.00). Importantly, when restricting the analysis to patients with pure phenotypes, SSEP abnormalities remained significantly more frequent in SCA3 (7 of 8 patients) than in SCA10 (2 of 15 patients) (*p* = 0.001).

**Fig. 1:**
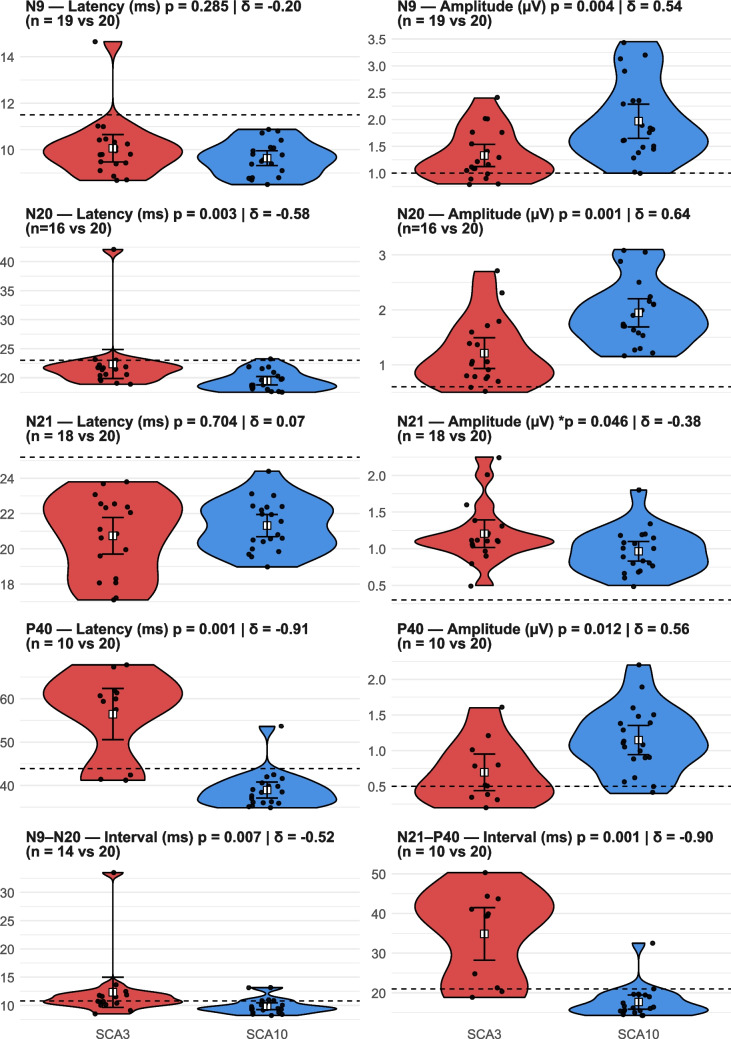
Violin plot for the latencies and amplitudes comparing the SCA3 and SCA10 groups. Legend: Quantitative analyses were performed using averaged bilateral values when available; when a response was absent on one side, the preserved side was used. Cases with bilateral absence were excluded from parameter-specific analyses. The dashed line represents the normal reference values. *After exclusion of outliers by the IQR method, the adjusted *p* value was = 0.054

After excluding absent potentials, we compared the quantitative values for mean latencies and amplitudes between the SCA3 and SCA10 groups, with data summarized in Fig. 1. In our cohort, SCA3 presented more prolonged N20, N9-20, P40, and N21-P40 latencies, and smaller N9, N20, and P40 amplitudes. Patients with SCA10 showed lower N21 amplitudes (Fig. 1) compared to SCA3 in the primary analysis (*p* = 0.046, δ =  − 0.39). However, in sensitivity analyses excluding outliers identified using the IQR method, this difference was no longer statistically significant (*p* = 0.054), with minimal change in effect size.

**Fig. 2:**
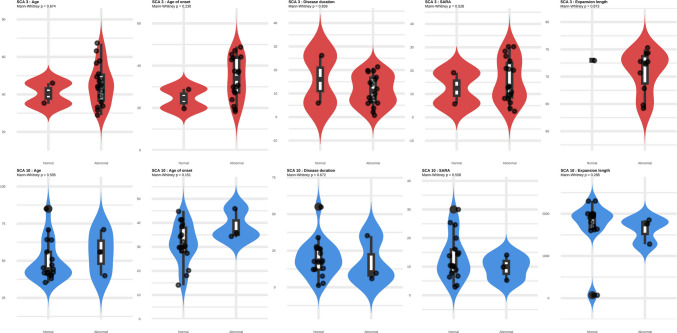
Violin plot for the demographic and clinical data distribution (Normal Vs Abnormal exam)

Regarding the association between demographic and clinical variables and SSEP abnormalities, there was no statistically significant difference in the distribution of the clinical variables between patients with normal SSEP exams and those with abnormal SSEP exams in either the SCA3 or SCA10 group (Fig. 2).

We further explored correlations between the clinical variables and SSEP abnormalities by fitting a penalized LASSO regression model. However, the high level of separation among the groups — with 18 patients with abnormal SSEP in the SCA3 group and 17 patients with normal SSEP in the SCA10 group — led to unstable estimates with all coefficients approaching zero. Although we employed Bootstrap resampling (B = 1000) to reduce the risk of overfitting and strengthen our analysis, this data is presented as supplementary material for exploratory and descriptive purposes only and should be interpreted with caution (Suppl Fig. 1).

The neuroimaging findings did not show any correlation with the presence of abnormalities in the cortical components of the SSEPs in the dichotomous normal vs. abnormal MRI classification (Fisher’s exact test = 1.00 for both groups). In the SCA3 group, no significant association was observed between GCA scores and SSEP abnormalities (Spearman’s rho = 0.06, *p* = 0.79). Similarly, in the SCA10 group, no association was found between GCA scores and SSEP abnormalities (Spearman’s rho = 0.03, *p* = 0.91).

## Discussion

The present study demonstrates distinct somatosensory evoked potential (SSEP) profiles between patients with SCA3 and SCA10. SSEP abnormalities were markedly more frequent in SCA3, predominantly involving central conduction and cortical responses, whereas most patients with SCA10 exhibited preserved neurophysiological patterns, even in the context of longstanding disease. The high statistical power observed for the primary outcome further supports the robustness of the group differences, suggesting that, despite the modest sample size, the observed group differences are unlikely to be explained by sampling variability alone and likely reflect a disease-specific pattern of somatosensory pathway involvement.

These findings support the concept that SCA3 is associated with more widespread involvement of somatosensory pathways, while SCA10, particularly in Southern Brazilian cohorts, tends to present with a more restricted phenotype predominantly affecting the cerebellum. The phenotypic distribution observed in our sample is consistent with previous regional studies [4, 12, 13].

In the SCA3 group, SSEP abnormalities predominantly reflected central conduction impairment, as evidenced by prolongation of interpeak latencies (N9–N20 and N21–P40) and frequent absence of cortical responses, particularly the P40 potential. In contrast, peripheral components were relatively preserved. Additionally, abnormalities were more prominent in latency than amplitude measures, suggesting a predominance of conduction slowing within the somatosensory pathways.

These findings are consistent with involvement of the dorsal-lemniscal pathway at central levels, in line with the known multisystem neurodegeneration observed in SCA3 [6–10, 28, 29]. Previous studies have reported SSEP abnormalities in SCA3, particularly following tibial nerve stimulation, with findings including delayed or absent cortical responses and prolonged central conduction times [18–21]. However, comparisons across studies are limited by methodological heterogeneity, including differences in stimulation protocols, components analyzed, and sample sizes.

In this context, our findings of frequent central conduction abnormalities and cortical involvement are consistent with prior neurophysiological data. Importantly, the high frequency of P40 abnormalities observed in our cohort reinforces the vulnerability of long ascending somatosensory pathways in SCA3, particularly those subserving lower limb afferent input. Rather than indicating a specific underlying mechanism, latency prolongation in this context likely reflects a combination of factors, including reduced fiber density, impaired temporal synchronization, and tract degeneration along the ascending sensory pathways [28–33]. Advanced neuroimaging techniques, including diffusion tensor imaging, have identified early abnormalities in the medial lemniscus even in pre-ataxic stages of SCA3, suggesting that somatosensory pathway involvement may occur early in the disease course [29]. Together, these findings provide a structural and functional framework that supports the neurophysiological abnormalities observed in our cohort.

An additional point of interest concerns the subgroup of SCA3 patients classified as having peripheral neuropathy. Among these, three patients had electrodiagnostic confirmation of axonal polyneuropathy, whereas one patient was classified based on clinical findings alone. Notably, these patients exhibited preserved peripheral SSEP components, with abnormalities predominantly affecting central conduction. Given that SSEPs are only able to assess the dorsal-lemniscal pathway, it is unclear if this discrepancy reflects the involvement of alternative peripheral pathways, captured by the NCS. For the patient without electrodiagnostic documentation, it is possible that the peripheral clinical manifestations reflect involvement of the peripheral nerve, such as the anterior and posterior horns of the spinal cord, the dorsal root ganglion, and the dorsal column, mimicking a peripheral neuropathy phenotype [6–10, 28–33].

Given the absence of systematic nerve conduction studies in all patients, the distinction between peripheral and central contributions to the sensory phenotype in this subgroup should be interpreted with caution. Future studies incorporating standardized electrodiagnostic assessment may help clarify the relative contribution of peripheral nerve involvement in SCA3.

These findings can also be interpreted in light of the reciprocal relationship between cerebellar circuitry and the somatosensory system [34]. Disruption of somatosensory afferent input may contribute to impaired cerebellar processing, while cerebellar dysfunction may, in turn, affect somatosensory cortical activity through altered sensorimotor integration and feedback mechanisms. In this context, cerebellar and somatosensory dysfunction may interact bidirectionally, contributing to the overall burden of disease in SCA3.

In contrast to SCA3, most patients with SCA10 exhibited preserved SSEP responses, supporting the concept that somatosensory pathways are relatively spared in this condition, particularly in Southern Brazilian cohorts. This finding is consistent with neuropathological and neuroimaging studies demonstrating predominant cerebellar involvement with relative preservation of extracerebellar structures in this population [7, 8, 35, 36].

A small subset of patients with SCA10 showed abnormalities affecting central conduction and cortical responses. Given the limited number of cases and the absence of detailed structural or functional imaging targeting somatosensory pathways in these individuals, these findings should be interpreted cautiously. Rather than reflecting a consistent disease feature, they may represent phenotypic variability or involvement of specific network-level mechanisms.

No abnormalities suggestive of peripheral nerve involvement were identified in the SCA10 group, which is consistent with the predominance of pure cerebellar ataxia in our cohort. The presence of peripheral neuropathy in SCA10 remains controversial. Although early descriptions of the disease reported peripheral nerve involvement [37], subsequent studies have not systematically evaluated this feature using standardized electrodiagnostic methods [32].

Recent multimodal neuroimaging studies have suggested alterations in functional connectivity within cerebellar and sensorimotor networks in SCA10, indicating that network dysfunction may extend beyond purely structural degeneration [14]. In this context, future studies combining SSEPs with advanced neuroimaging techniques may help clarify whether such network-level alterations contribute to somatosensory pathway involvement in selected cases.

The phenotypic profile observed in our SCA10 cohort, characterized by a predominance of pure cerebellar ataxia and low frequency of extracerebellar features, is consistent with previous reports from Southern Brazil [11–13, 38]. This contrasts with Hispanic-Amerindian populations, in which epilepsy and broader multisystem involvement are more frequent.

These population-specific differences may have implications for the extent of somatosensory pathway involvement and should be considered when interpreting and generalizing neurophysiological findings across different SCA10 cohorts. Replication of SSEP studies in populations with distinct genetic backgrounds may help clarify the variability of sensory pathway involvement in this disorder.

No significant associations were identified between SSEP abnormalities and demographic or clinical variables in either group. These findings were consistent across univariate analyses, suggesting that the presence of neurophysiological abnormalities is not directly explained by disease duration, clinical severity, or other measured variables.

Although a penalized LASSO regression model was explored, the marked separation between groups and the limited sample size resulted in model instability. Therefore, these results should be considered exploratory and are presented for descriptive purposes only. Overall, our findings support a relative dissociation between clinical phenotype and somatosensory pathway involvement in these conditions, as previously suggested in spinocerebellar ataxias [33].

No significant associations were observed between neuroimaging findings and SSEP abnormalities in either group. In particular, GCA scores did not correlate with the presence of neurophysiological alterations, suggesting a dissociation between global cortical atrophy and somatosensory pathway dysfunction.

This finding may reflect the fact that SSEPs predominantly assess the functional integrity of specific ascending sensory pathways, whereas visual rating scales such as GCA capture more diffuse and nonspecific patterns of cortical atrophy. As such, structural changes detectable on conventional MRI may not directly correspond to functional impairment within the dorsal-lemniscal pathway. In this context, advanced neuroimaging approaches, including volumetric analysis and functional connectivity studies, may provide more sensitive markers of the relationship between structural and neurophysiological changes.

This study has several limitations. First, its cross-sectional design precludes assessment of the temporal evolution of SSEP abnormalities. Longitudinal studies would be valuable to determine whether such abnormalities emerge or progress over time, particularly in SCA10 patients.

Second, the modest sample size and single-center recruitment may limit the generalizability of our findings. Although the difference between groups was marked and supported by a large effect size, the sample size may be insufficient for more complex analyses, including subgroup comparisons and multivariable models, such as the LASSO regression analysis.

Third, the absence of a healthy control group represents an important limitation. SSEP abnormalities were defined based on established normative reference values; however, these may vary across laboratories and may not fully capture interindividual variability. In particular, the low frequency of abnormalities observed in SCA10 should be interpreted with caution, as it is not possible to determine whether these findings exceed normal variation in the absence of matched controls.

Fourth, the study was not conducted under blinded conditions, as examiners were aware of the patients’ genetic diagnoses at the time of SSEP acquisition and interpretation. Although SSEPs are relatively objective measures with standardized waveform identification, the possibility of unconscious bias cannot be entirely excluded. Future studies should consider blinded analysis or independent review of neurophysiological data. Although height was assessed during SSEP examination it was not included as a variable for further analysis, representing another study limitation.

Finally, although all patients had genetically confirmed diagnoses, nearly half of the SCA3 cohort lacked data on CAG repeat length. This reflects structural limitations in access to detailed molecular testing, as some genetic reports obtained through institutional collaborations confirm the presence of an expanded allele without specifying repeat size. We acknowledge this limitation, which is not uncommon in centers operating in resource-limited settings [39]. Sensitivity analyses did not identify significant differences between patients with and without available genetic data, suggesting that missingness is unlikely to have introduced substantial bias.

Future studies should aim to further characterize somatosensory pathway involvement in spinocerebellar ataxias through larger, multicenter cohorts and longitudinal designs. In particular, the inclusion of age- and sex-matched healthy control groups will be essential to better define the magnitude and clinical relevance of SSEP abnormalities, especially in conditions such as SCA10, where alterations appear to be infrequent.

The integration of SSEPs with advanced neuroimaging techniques represents a promising avenue for future research. Quantitative MRI approaches, including volumetric analyses and diffusion tensor imaging, may help clarify the relationship between structural degeneration of the dorsal column–medial lemniscus system and functional impairment. In addition, functional neuroimaging studies may provide insights into network-level alterations that are not captured by conventional structural imaging.

Further investigation into genotype–phenotype correlations is also warranted. In SCA3, systematic assessment of CAG repeat length in larger samples may help determine whether somatosensory pathway involvement relates to genetic burden. In SCA10, studies exploring the role of repeat expansion architecture and its association with phenotypic variability may help explain the heterogeneity of neurophysiological findings [40–45].

Finally, multimodal neurophysiological approaches combining SSEPs with other techniques, such as motor evoked potentials and peripheral nerve conduction studies, may provide a more comprehensive understanding of the distribution of central and peripheral involvement in these disorders.

In conclusion, our findings demonstrate distinct neurophysiological profiles between SCA3 and SCA10, highlighting the heterogeneity among spinocerebellar ataxias. SSEP abnormalities were markedly more frequent in SCA3 and predominantly reflected involvement of central somatosensory pathways, whereas most patients with SCA10 exhibited preserved responses, suggesting relative pathway integrity in this population. No significant associations were identified between SSEP abnormalities and demographic, clinical, imaging, or genetic variables, supporting a relative dissociation between clinical phenotype and somatosensory pathway dysfunction.

These findings provide a neurophysiological framework for understanding sensory pathway involvement in different forms of spinocerebellar ataxia and may serve as a basis for future multimodal studies integrating electrophysiology and advanced neuroimaging approaches.

## Supplementary Information

Below is the link to the electronic supplementary material.

Supplementary file1 (PDF 285 KB)

Supplementary file2 (DOCX 17 KB)

## Data Availability

Data available on request from the authors.
