# Supplementary figures and images for: Somatosensory Evoked Potentials in Spinocerebellar Ataxia Type 3 and Type 10
Source: Cerebellum. 2026 Apr 17;25(3):57. doi: 10.1007/s12311-026-01998-0 (PMC13090235; doi:10.1007/s12311-026-01998-0)

LASSO penalized regression with bootstrap resampling (B = 1000)  
Comparison between SCA3 and SCA10

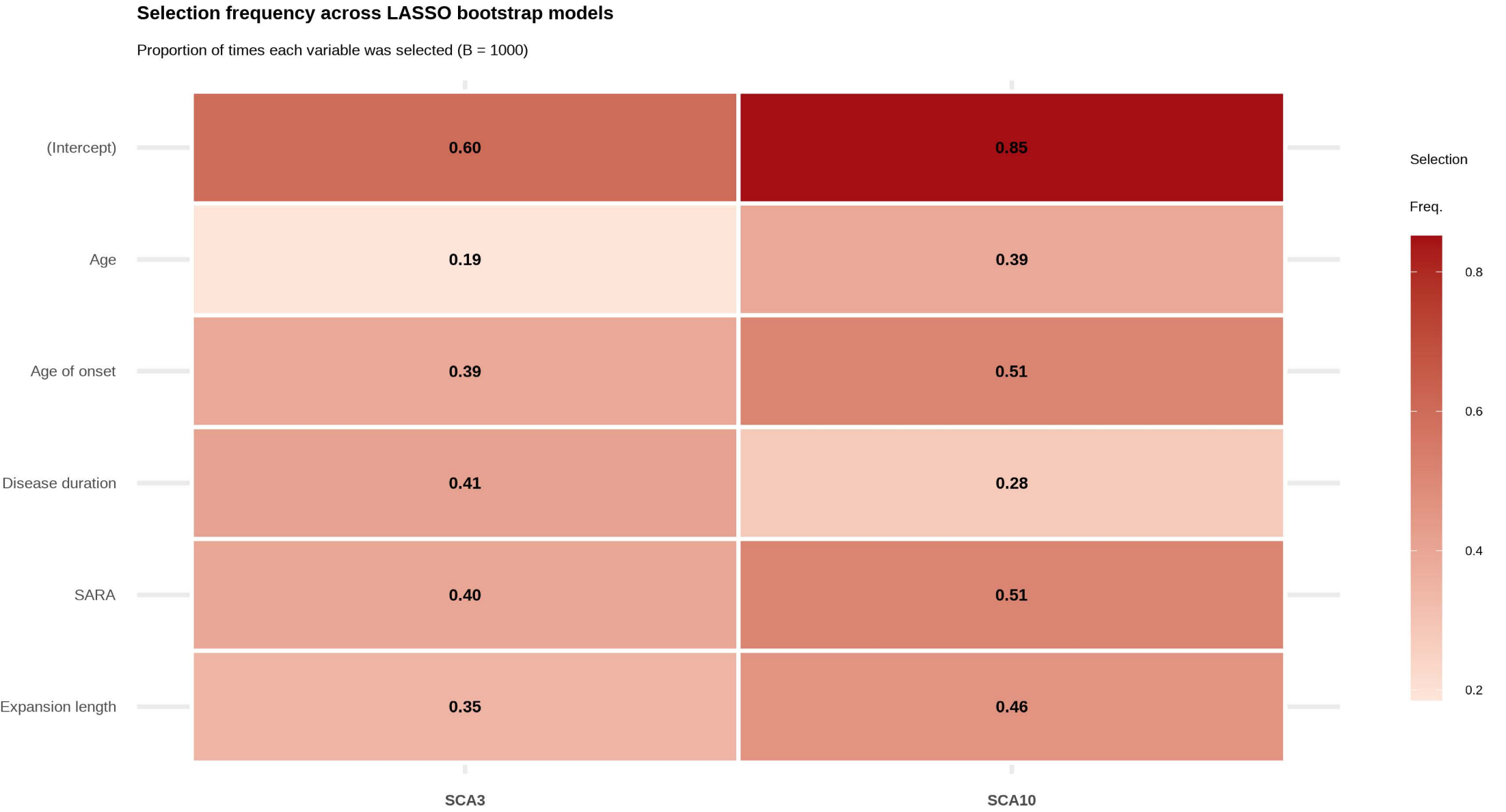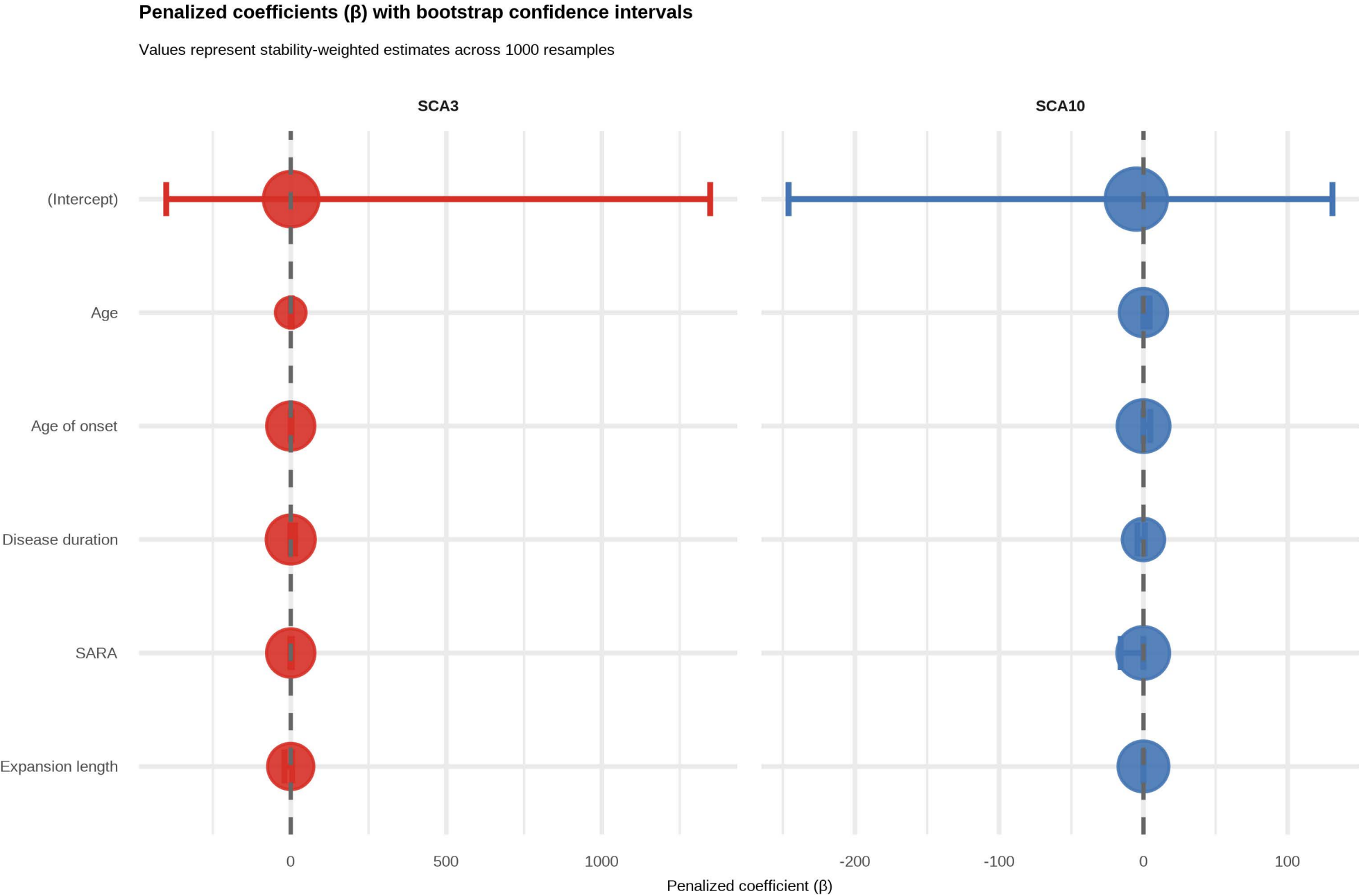

Supplement: Supplementary file 1 — Supplementary file1 (PDF 285 KB) [file 12311_2026_1998_MOESM1_ESM.pdf]
