# Supplementary material for: Somatosensory Evoked Potentials in Spinocerebellar Ataxia Type 3 and Type 10
Source: Cerebellum. 2026 Apr 17;25(3):57. doi: 10.1007/s12311-026-01998-0 (PMC13090235; doi:10.1007/s12311-026-01998-0)
Supplement: Supplementary file 2 — Supplementary file2 (DOCX 17 KB) [file 12311_2026_1998_MOESM2_ESM.docx]

**Supplementary table 1:** Normative reference values and criteria for SSEP interpretation based on IFCN guidelines [16].

| **MEDIAN NERVE SSEP** | | | | | | | | | |
| --- | --- | --- | --- | --- | --- | --- | --- | --- | --- |
| **Parameter** | | **Mean** | | **ULN or LLN (mean ± 3SD)** | | **Side-to-side difference** | | **Definition of abnormality** | |
| **Latency (ms)** | | | | | | | | | |
| N9 | | 9.8 | | 11.5 | | - | | > ULN | |
| N20 | | 19.8 | | 23.0 | | 1.4 | | > ULN or asymmetry | |
| **Interpeak interval (ms)** | | | | | | | | | |
| N9-N20 | | 9.3 | | 10.8 | | 0.9 | | > ULN or asymmetry | |
| **Amplitude (µv)** | | | | | | | | | |
| N9 | | 4.8 | | 1.0 | | 50% | | < LLN or asymmetry | |
| N20 (baseline to peak) | | 2.2 | | 0.6 | | 50% | | < LLN or asymmetry | |
| **TIBIAL NERVE SSEP** | | | | | | | | | |
| **Parameter** | **Mean** | | **ULN or LLN (mean ± 3SD)** | | **Side-to-side difference** | | **Height correction (if < or > 175cm)** | | **Definition of abnormality** |
| **Latency (ms)** | | | | | | | | | |
| N21 | 21.8 | | 25.2 | | 1.1 | | ± 0.18 ms/cm | | > ULN (height adjusted) |
| P40 | 38.0 | | 43.9 | | 2.1 | |  |  | > ULN (height adjusted) |
| **Interpeak interval (ms)** | | | | | | | | | |
| N21-P40 | 16.0 | | 21.0 | | 2.1 | | - | | > ULN |
| **Amplitude (µv)** | | | | | | | | | |
| N21 | 1.1 | | 0.3 | | - | | - | | < LLN |
| P40 | 1.8 | | 0.5 | | - | | - | | < LLN |
| **CRITERIA FOR ABNORMALITY** | | | | | | | | | |
| **Prolonged latency / interval** | | | | | Value exceeding the upper limit of normal (ULN) | | | | |
| **Reduced amplitude** | | | | | Value below the lower limit of normal (LLN) | | | | |
| **Absent response** | | | | | No reproducible waveform despite adequate stimulation and recording conditions | | | | |

Reference values correspond to typical adult populations (mean height approximately 1.70 m). Latency values for tibial nerve SSEPs were interpreted considering body height due to known physiological dependence on limb length. ULN: upper limit of normal; LLN: lower limit of normal.

*N21 and P40 correspond to the lumbar and cortical components of tibial nerve SSEPs, respectively, as defined in the study protocol.
